# Loss of Sarm1 does not suppress motor neuron degeneration in the SOD1^G93A^ mouse model of amyotrophic lateral sclerosis

**DOI:** 10.1093/hmg/ddy260

**Published:** 2018-07-14

**Authors:** Owen M Peters, Elizabeth A Lewis, Jeannette M Osterloh, Alexandra Weiss, Johnny S Salameh, Jake Metterville, Robert H Brown, Marc R Freeman

**Affiliations:** 1 Department of Neurobiology, University of Massachusetts Medical School, Worcester, MA, USA; 2 Department of Neurology, University of Massachusetts Medical School, Worcester, MA, USA

## Abstract

Axon degeneration occurs in all neurodegenerative diseases, but the molecular pathways regulating axon destruction during neurodegeneration are poorly understood. Sterile Alpha and TIR Motif Containing 1 (Sarm1) is an essential component of the prodegenerative pathway driving axon degeneration after axotomy and represents an appealing target for therapeutic intervention in neurological conditions involving axon loss. Amyotrophic lateral sclerosis (ALS) is characterized by rapid, progressive motor neuron degeneration and muscle atrophy, causing paralysis and death. Patient tissue and animal models of ALS show destruction of upper and lower motor neuron cell bodies and loss of their associated axons. Here, we investigate whether loss of Sarm1 can mitigate motor neuron degeneration in the SOD1^G93A^ mouse model of ALS. We found no change in survival, behavioral, electrophysiogical or histopathological outcomes in SOD1^G93A^ mice null for Sarm1. Blocking Sarm1-mediated axon destruction alone is therefore not sufficient to suppress SOD1^G93A^-induced neurodegeneration. Our data suggest the molecular pathways driving axon loss in ALS may be Sarm1-independent or involve genetic pathways that act in a redundant fashion with Sarm1.

## Introduction

Axon degeneration is a hallmark of neurodegenerative disease (1), but we lack a detailed understanding of the signaling pathways that promote this destructive process in any major disorder. Significant progress has however been made in the characterization of molecules that regulate axon destruction after axotomy, termed Wallerian degeneration (2). Initial molecular insights into Wallerian degeneration came from the spontaneous mouse mutant *Wallerian degeneration slow* (*Wld^S^*) (3). Remarkably, while severed axons of wild-type mice typically undergo destruction and clearance within days, *Wld^S^* mutant axons remain intact for weeks after lesion. This neuroprotective effect of *Wld^S^* mapped to a genome rearrangement producing a neomorphic fusion protein (4), with the key protective activity determined to be that of Nicotinamide adenine dinucleotide (NAD^+^) biosynthetic enzyme nicotinamide mononucleotide adenylyltransferase 1 (Nmnat1) (5). The *Wld^S^* mutant radically changed our views on axon biology—demonstrating that axon destruction could be regulated and that under some conditions axons could suvive for long periods without a cell body.

Is there an endogenous pathway that actively drives axon auto-destruction? An unbiased forward-genetic screen in *Drosophila* for mutants that phenocopied *Wld^S^* led to the discovery of the Sterile Alpha and TIR Motif Containing 1 (dSarm/Sarm1), a founding member of the axon death signaling pathway engaged after axotomy (6). In *dsarm* null mutant backgrounds, distal severed axons survived for weeks post-axotomy, an effect that was found to be conserved after sciatic nerve lesion in mice (6,7). Moreso, as the Sarm1 protein is highly conserved between humans and rodents (Human accession number: Q6SZW1-1, Mouse accession number Q6PDS3-1; 94% identity, 99% coverage), functional conservation is likely. At least two additional genes have subsequently been identified as similarly potent regulators of axon degeneration: the E3 ubiquitin ligase Phr1/Highwire (8–10) and the bric-graveà-brac, tramtrack, broad complex (BTB)/BTB and C-terminal Kelch (BACK) domain containing gene Axundead (11).

Given that each of these molecules are essential for efficient axon degeneration after axotomy, they represent exciting new potential targets for therapeutic blockade of axon loss in disease. The mitigating activity of *Wld^S^* has previously been assessed in models of toxin-induced neuropathies (12,13), gracile axonal dystrophy (14), glaucoma (15,16), Parkinson’s disease (17,18) and motor neuron diseases including amyotrophic lateral sclerosis (ALS) (19–21) and met with some success, suggesting that treating the axon is a viable approach. The discovery of Sarm1 and other axon death signaling molecules warrants a reassessment of the role of axon degeneration pathways in neurodegenerative diseases. Indeed, a recent study has demonstrated that the deletion of Sarm1 is in fact significantly more protective than Wld^S^ expression in an Nmnat2 depletion model of neurodegeneration (22).

ALS exists in idiopathic and less common familial forms and is characterized by progressive paralysis, a consequence of profound destruction of motor neurons and their axons (23). Notably, Single nucleotide polymorphisms associated with Sarm1 have been detected in genome-wide association study of sporadic ALS patients, suggesting the gene may even play a direct role in ALS pathogenesis (24). In this study we tested whether elimination of Sarm1 was sufficient to block axon destruction by the human SOD1^G93A^ mutant molecule in the transgenic mouse model of ALS. We found that loss of Sarm1 was sufficient to block Wallerian degeneration in 1-year-old control mice, and at early stages in transgenic mice expressing mutant SOD1. However, elimination of Sarm1 was not sufficient to mitigate behavioral, morphological or electrophysiological deficits observed in motor neurons in the ALS mouse. Our findings indicate the molecular pathways driving axon loss in the SOD^G93A^ model may be Sarm1-independent or that in the context of disease Sarm1 acts in a redundant fashion with other pro-degenerative signaling pathways.

## Results

### Degeneration of severed axons in aged mice is ameliorated by loss of Sarm1

**Figure 1:**
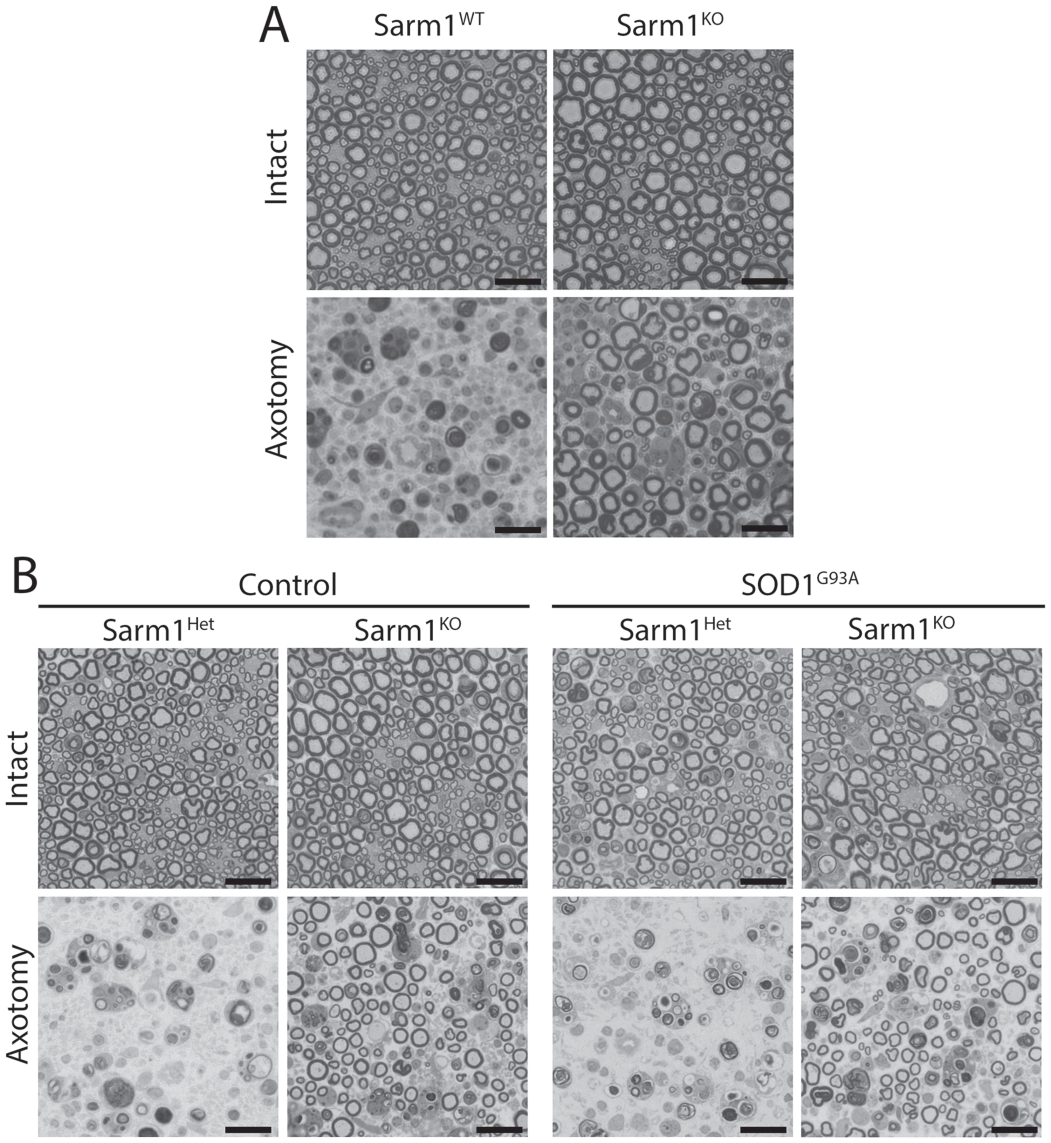
Sarm1-mediated axon destruction can be suppressed in aged mice and is not altered by transgenic expression of mutant SOD1. (**A**) Representative micrographs showing toluidine blue-stained intact or axotomized sciatic nerves of 1-year-old wild-type Sarm1^KO^ mice. Morphologically intact axons were widespread in the distal sciatic nerve 14-day post-lesion in Sarm1^KO^ mice. (**B**) Representative images of intact or axotomized sciatic nerve of 5-week-old control and SOD1^G93A^ transgenic mice 14 days post-axotomy, showing preservation of axons in Sarm^KO^ mice. Scale bar = 20 μm.

The paralytic symptoms of ALS result from the progressive degeneration of upper and lower motor neurons and their axons, with typical disease onset in adulthood and risk increasing with age. We have previously demonstrated that when severed *in vivo*, peripheral nerve axons of young Sarm1 knock out (Sarm1^KO^) mice are robustly protected against Wallerian degeneration (6). For Sarm1 to be a viable target for treatment of ALS or other late-onset neurodegenerative diseases this mechanism of programmed axon destruction must be maintained in aged neurons. To test this, we lesioned the sciatic nerves of 1-year-old Sarm1^KO^ mice and inspected the morphology of myelinated motor and sensory axons in the distal, severed nerve portion 14 days post-lesion (Fig. 1A). Consistent with young animals the nerves of aged wild-type control mice were in a highly degenerate state, with normal myelinated axons absent. In Sarm1^KO^ mice, however, a substantial proportion of axons retained normal morphology, included being enclosed by an intact myelin sheath. Protection at this mature adult stage demonstrates that, rather than being a mechanism retained from development in young adult neurons, Sarm1-mediated axon destruction is an intrinsic characteristic of post-developmental neurons that may contribute to neurodegeneration and injury.

### Mutant SOD1^G93A^ does not impede protection against Wallerian degeneration in Sarm1 null nerves

SOD1^G93A^ transgenic mice express high levels of mutant SOD1 protein throughout their nervous system (25). It was important to confirm that excessive levels of this protein did not interfere with normal Sarm1-mediated Wallerian degeneration. Sciatic nerve axotomy experiments were conducted in young SOD1^G93A^ transgenic mice that were Sarm1 heterozygous (Sarm1^Het^), where axotomy phenotype is as wild type or null for Sarm1 (Fig. 1B). At 14 days post-lesion myelinated axons of Sarm1^Het^ mice expressing SOD1^G93A^ underwent axon degeneration comparable to that observed in control Sarm1^Het^ and aged Sarm1^WT^ (Fig. 1A) animals. Mutant SOD1 transgenic axons in Sarm1^KO^ mice were comparably protected from Wallerian degeneration to those of Sarm1^KO^ control mice, confirming that the human SOD1^G93A^ transgene does not impede protection of severed axons.

### Blocking Sarm1-mediated axon destruction does not alter the acquisition of motor defects in the SOD1^G93A^ transgenic mouse

**Figure 2:**
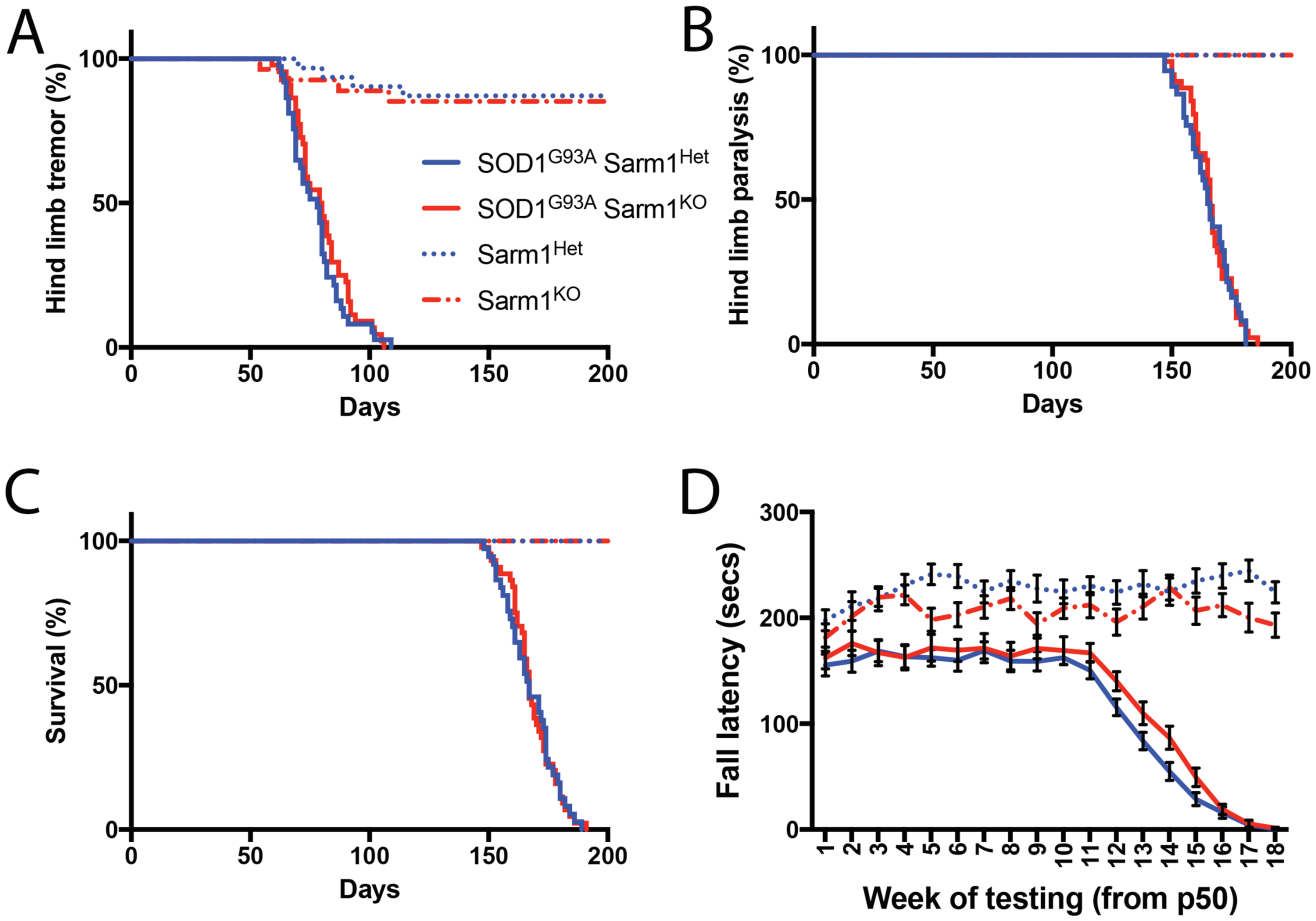
Sarm1 does not contribute to onset, survival or sensorimotor performance deficits in SOD1^G93A^ mice. Percentage of transgenic colony free of (**A**) hind limb tremor and (**B**) hind limb paralysis. (**C**) Kaplan–Meyer plot showing survival of transgenic cohorts (**A**–**C**: Sarm1^Het^ SOD1^G93A^, n = 37; Sarm1^KO^ SOD1^G93A^, n = 44; Sarm1^Het^ control, n = 31; Sarm1^KO^ control, n = 27; Sarm1^Het^ SOD1^G93A^ vs Sarm1^KO^ SOD1^G93A^; Log-Rank test, *P*-value > 0.05). (**D**) Fall latency of male transgenic mice on 4–40 rpm accelerating rotarod task for assessment of sensorimotor function (SOD1^G93A^ groups, n = 20; control groups, n = 15; two-way ANOVA with Bonferonni post-hoc test).

Having confirmed Sarm1-mediated Wallerian degeneration occurs normally in the SOD1^G93A^ transgenic mouse, we next tested if this mechanism contributes to the chronic neurodegenerative and subsequent paralytic phenotype developed by these animals (Fig. S1). We generated a colony of SOD1^G93A^ transgenic mice either heterozygous for Sarm1, where Wallerian degeneration occurs normally, or homozygous null for Sarm1 where distal severed axons are preserved (Fig. 1B). Mice were blindly scored daily for onset of tremor at early stages of disease and onset of paralysis of the hind limbs at later stages (Fig. 2A,B). There was no significant difference in the presentation of either of these symptoms between Sarm1 heterozygous or null SOD1^G93A^ transgenic mice (Sarm1^Het^ SOD1^G93A^, n = 37; Sarm1^KO^ SOD1^G93A^, n = 44. Tremor onset: Sarm1^Het^ SOD1^G93A^, 78.0; Sarm1^KO^ SOD1^G93A^, 79.5. Log-Rank (Mantel–Cox test): *χ*^2^ = 0.8999, *df* = 1, *P*-value = 0.3428. Hind limb paralysis onset: Sarm1^Het^ SOD1^G93A^, 165.0; Sarm1^KO^ SOD1^G93A^, 166.0. Log-Rank (Mantel–Cox test): *χ*^2^ = 0.1047, *df* = 1, *P*-value = 0.7462). Furthermore, no difference was detected in lifespan between SOD1^G93A^ transgenic mice heterozygous or null for Sarm1 (Fig. 2C) (Sarm1^Het^ SOD1^G93A^, n = 37; Sarm1^KO^ SOD1^G93A^, n = 44; Sarm1^Het^ SOD1^G93A^, 167.0; Sarm1^KO^ SOD1^G93A^, 167.0. Log-Rank (Mantel–Cox test): *χ*^2^ = 0.005961, *df* = 1, *P*-value = 0.9385). We next assayed for potential rescue of sensorimotor function in the Sarm1^KO^ background more quantitatively by using rotarod (Fig. 2D) and grip strength testing (Fig. S2). Though SOD1^G93A^ expression resulted in progressive and significant deline in motor performance post-hoc tests did not detect any significant changes in sensorimotor defects associated with SOD1^G93A^ in the Sarm1 null background compared to sibling heterozygous controls in either rotarod (SOD1^G93A^ groups, n = 20; control groups, n = 15; F_Interaction_ (34 864) = 11.75,*P*-value < 0.0001; F_Timepoint_ (17 864) = 35.34, *P*-value < 0.0001; F_Genotype_ (2864) = 559.2, *P*-value < 0.0001; Bonferroni post-hoc analysis detected no significant differences between SOD1^G93A^ transgenic groups) or grip strength test (Fig. S2). These data indicate that elimination of Sarm1 is not sufficient to delay the onset or progression of the chronic motor deficits in the SOD1^G93A^ mice.

### ALS-associated deficits in neuromuscular electrophysiological function are not protected by loss of Sarm1

Although removed from neuronal circuits through loss of their cell body and dendrites, severed axons protected from Wallerian degeneration are able to maintain electrophysiological function and evoke action potentials in response to artificial stimulation (4,11). It was thus feasible that despite not ameliorating the overall ALS-like phenotypes of SOD1^G93A^ mouse, Sarm1 null motor neurons may preserve their total number and size of their motor units and maintain their ability to conduct action potentials relative to SOD1^G93A^ mice expressing Sarm1.

**Figure 3:**
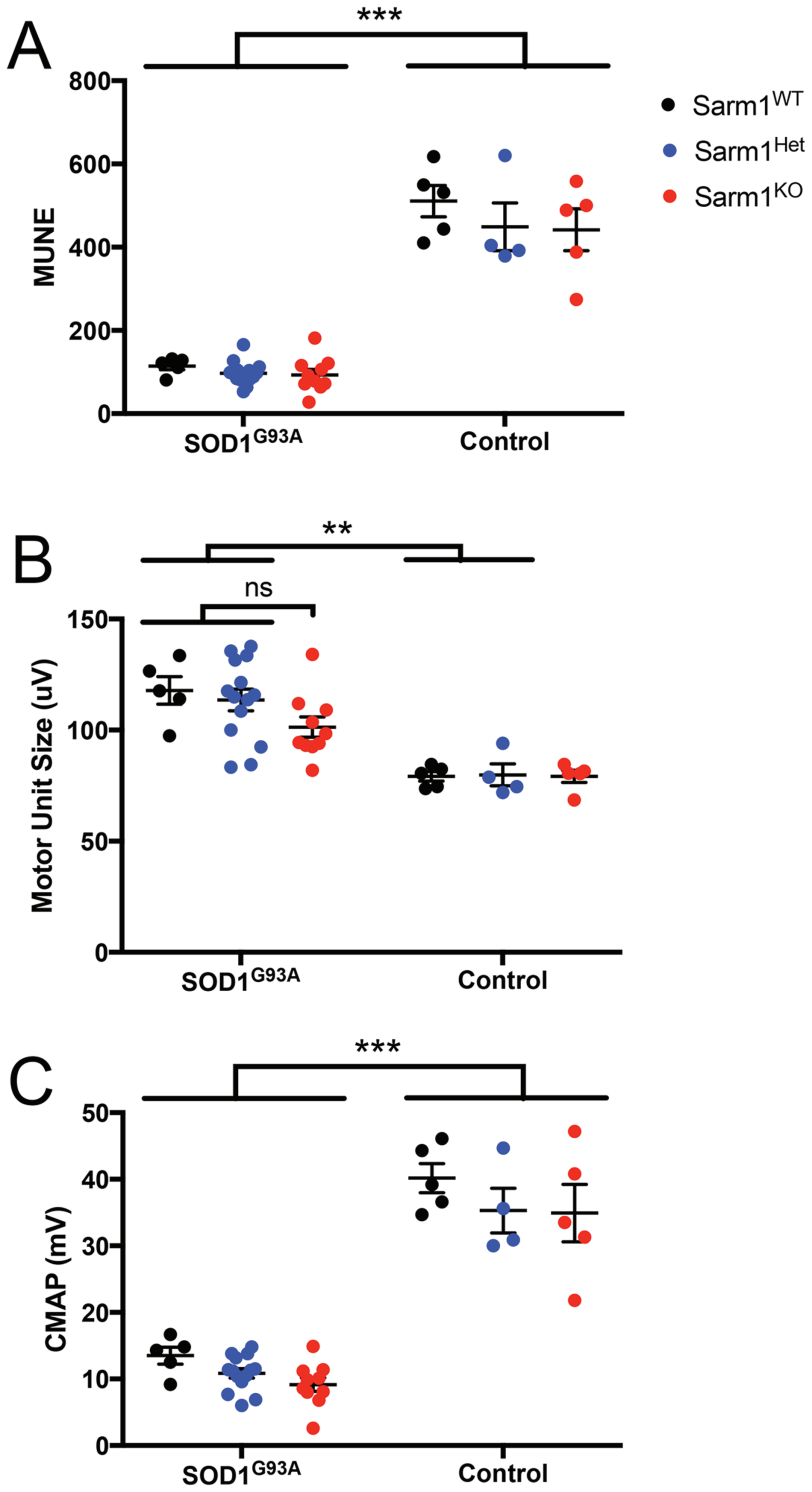
Deficits in neuromuscular electrophysiological function associated with SOD1^G93A^ expression are not ameliorated in mice lacking Sarm1. Neuromuscular electrophysiological function was tested in SOD1^G93A^ and control mice of Sarm1^WT^ (black), Sarm1^Het^ (blue) or Sarm1^KO^ (red) genotype. Assessing the gastrocnemius muscle of p120–130 mice for (**A**) MUNE, (**B**) motor unit size and (**C**) CMAP failed to show any significant difference in phenotype between SOD1^G93A^ transgenic groups (mean ±  SEM, n = 4–14; two-way ANOVA with Bonferonni post-hoc test; ns = non-significant; **P-value < 0.01; ***P-value < 0.001).

To explore this possibility we used motor unit number estimation (MUNE) (26) to quantify several features of electrophysiological functional decline in the gastrocnemius muscle of our experimental cohorts (Fig. 3,S3). MUNE gives an estimation of the number of intact motor units present in a stimulated muscle, providing a robust read-out of neuromuscular electrophysiological decline in both ALS patients and rodent models (27,28). Assessment of adult control mice wild-type, heterozygous or homozygous null for Sarm1 detected no significant difference in the MUNE score. Therefore, loss of Sarm1 does not affect normal neuromuscular electrophysiological function as measured by MUNE (Fig. 3A). However, in p120–130 mice expressing the SOD1^G93A^ transgene we found a significant reduction in MUNE score (n = 4–14; two-way analysis of variance (ANOVA): F_Sarm1Genotype_ (2,37) = 1.692, *P*-value = 0.1981; F_SOD1Genotype_ (1,37) = 301.4, *P*-value < 0.0001; Bonferonni post-hoc test, ***P-value < 0.001). Loss of Sarm1 did not have a significant effect at this point nor at the early symptomatic p80–90 stage (Fig. S3). As MUNE score declines in ALS, a compensatory increase in motor unit size is seen with in both patients (28) and rodent models (27). Expression of SOD1^G93A^ indeed resulted in an increased motor unit size in controls, which was also present in all Sarm1 genotypes at p120–130 when compared to their littermate controls not expressing the SOD1^G93A^ transgene, although this trend was not statistically significant in the Sarm1^KO^ cohort (Fig. 3B). Post-hoc analysis between SOD1^G93A^ transgenic mice of differing Sarm1 genotypes revealed no significant difference between motor unit size in Sarm1 genotypes at p120–130 (n = 4–14; two-way ANOVA: F_Sarm1Genotype_ (2,37) = 1.148, *P*-value = 0.3283; F_SOD1Genotype_ (1,37) = 43.48, *P*-value < 0.0001; Bonferonni post-hoc test, **P-value < 0.01) or the earlier p80–90 time point (Fig. S3). Changes in motor unit size are a result of plasticity at the neuromuscular junction (NMJ) synapse. These data reveal that loss of Sarm1 does not play a biologically significant role in protecting motor unit size from increases in the context of the SOD^G93A^ model, arguing that loss of Sarm1 does not affect major aspects of synaptic plasticity. Finally, we assessed compound motor action potential (CMAP), a measure of total activity from synchronized action potentials reaching the muscle following simultaneous stimulation of multiple axons within a bundle. As with MUNE, total CMAP within the gastrocnemius was significantly reduced in mice expressing SOD1^G93A^. In control mice, the total CMAP was not altered in Sarm1^KO^ animals compared to Sarm1^Het^ or Sarm1^WT^, indicating loss of Sarm1 does not alter CMAP properties; however, the absence of Sarm1 did not ameliorate the deficits in CMAP observed in the presence of SOD^G93A^ (Fig. 3C; n = 4–14; two-way ANOVA: F_Sarm1Genotype_ (2,37) = 3.152, *P*-value = 0.0545; F_SOD1Genotype_ (1,37) = 260.5, *P*-value < 0.0001; Bonferonni post-hoc test, ***P-value < 0.001). Together, these data reveal that overall neuromuscular properties are relatively normal in Sarm1^KO^ animals,and that blockade of Sarm1 signaling alone is not sufficient to alter the decline in electrophysiological function of motor neurons in SOD1^G93A^ mice.

### Loss of Sarm1 does not preserve motor neuron axons or terminals in SOD1^G93A^ transgenic mice

**Figure 4:**
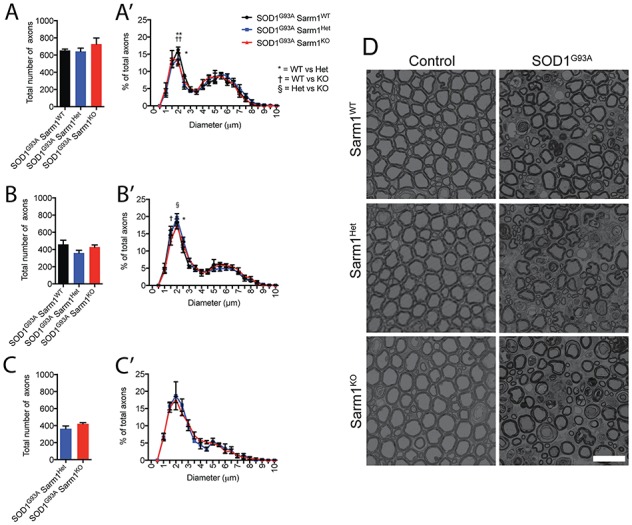
Progressive loss of myelinated motor axons is not ameliorated by loss of Sarm1. Total number of myelinated axons in the L5 ventral nerve root of SOD1^G93A^ transgenic mice wild type, heterozygous-null or homozygous-null for Sarm1 at (**A**) p80–90, (**B**) p120–130 and (**C**) end stage (n = 3–11, mean ± SEM; **A** and **B**, one-way ANOVA; **C**, Student *t* test). Frequency distribution of axon diameters at (**A′**) p90, (**B′**) p120 and (**C′**) endpoint expressed as percentage of total axons measured (mean ± SEM, two-way ANOVA with Bonferonni post-hoc test, *P-value < 0.05, **P-value < 0.01). (**D**) Representative images of L5 ventral nerve root myelinated axons at p120 in SOD1^G93A^ mice wild-type, heterozygous-null or homozygous-null for Sarm1 with comparison images from littermate controls not expressing SOD1^G93A^ (scale bar = 20 μm).

Neurodegeneration occurs throughout motor neurons of SOD1^G93A^ transgenic mice, with the cell body, axons and NMJs all being lost during the progression of pathology (29). Interestingly, these neurodegenerative mechanisms appear to be somewhat compartmentalized: suppression of apoptosis in SOD1 transgenic mice preserves spinal cord motor neuron cell bodies but does not rescue motor axons nor disease progression (30). Although we found that loss of Sarm1 failed to ameliorate any of the behavioral and physiological phenotypes of SOD1^G93A^ transgenic mice, it was conceivable that motor axons might remain morphologically intact with pathology being driven by degeneration of cell bodies or dendrites rendering the cells inactive. To determine whether axons were preserved we first quantified the number of myelinated motor axons with normal morphology present within the L5 ventral nerve root mutant SOD1^G93A^ transgenic cohorts at early, mid and end stages of pathology. When compared to transgenic mice carrying Sarm1, we found that no significant differences in number of healthy axons were seen in Sarm1 null mice at any stage in pathology (Fig. 4A–C: p90 SOD1^G93A^ Sarm1^WT^, n = 4; SOD1^G93A^ Sarm1^Het^, n = 4; SOD1^G93A^ Sarm1^KO^, n = 3. One-way ANOVA: F_Sarm1Genotype_ (2,8) = 1.133, *P*-value = 0.3688; p120–130 SOD1^G93A^ Sarm1^KO^, n = 4; SOD1^G93A^ Sarm1^Het^, n = 11; SOD1^G93A^ Sarm1^KO^, n = 7. One-way ANOVA: F_Sarm1Genotype_ (2,19) = 2.272, *P*-value = 0.1304; end-stage SOD1^G93A^ Sarm1^Het^, n = 3; SOD1^G93A^ Sarm1^KO^, n = 6. Unpaired two-tailed *t* test: *t* = 1.974, *df* = 7, *P*-value = 0.089). Large caliber Aα motor axons typically degenerate earlier than smaller fibers in the SOD1^G93A^ mouse, suggesting that some pools of fibers are inherently more vulnerable to the neurodegenerative insults associated with ALS. We therefore assessed the diameter of remaining healthy axons within the ventral nerve root, however found the distribution of fiber caliber to be consistent between SOD1^G93A^ transgenic cohorts regardless of Sarm1 genotype (Fig. 4A′–C′: p90 two-way ANOVA: F_Sarm1Genotype_ (2, 160) = 0.0004591, *P*-value = 0.9995; Bonferonni post-hoc test, *P-value < 0.05, **P-value < 0.01; p120 two-way ANOVA: F_Sarm1Genotype_ (2, 380) = 0.0001358, *P*-value = 0.9999; Bonferonni post-hoc test, *P-value < 0.05, **P-value < 0.01; endpoint two-way ANOVA: F_Sarm1Genotype_ (1, 140) = 0.0008991, *P*-value = 0.9761; Bonferonni post-hoc test, *P-value < 0.05, **P-value < 0.01).

**Figure 5:**
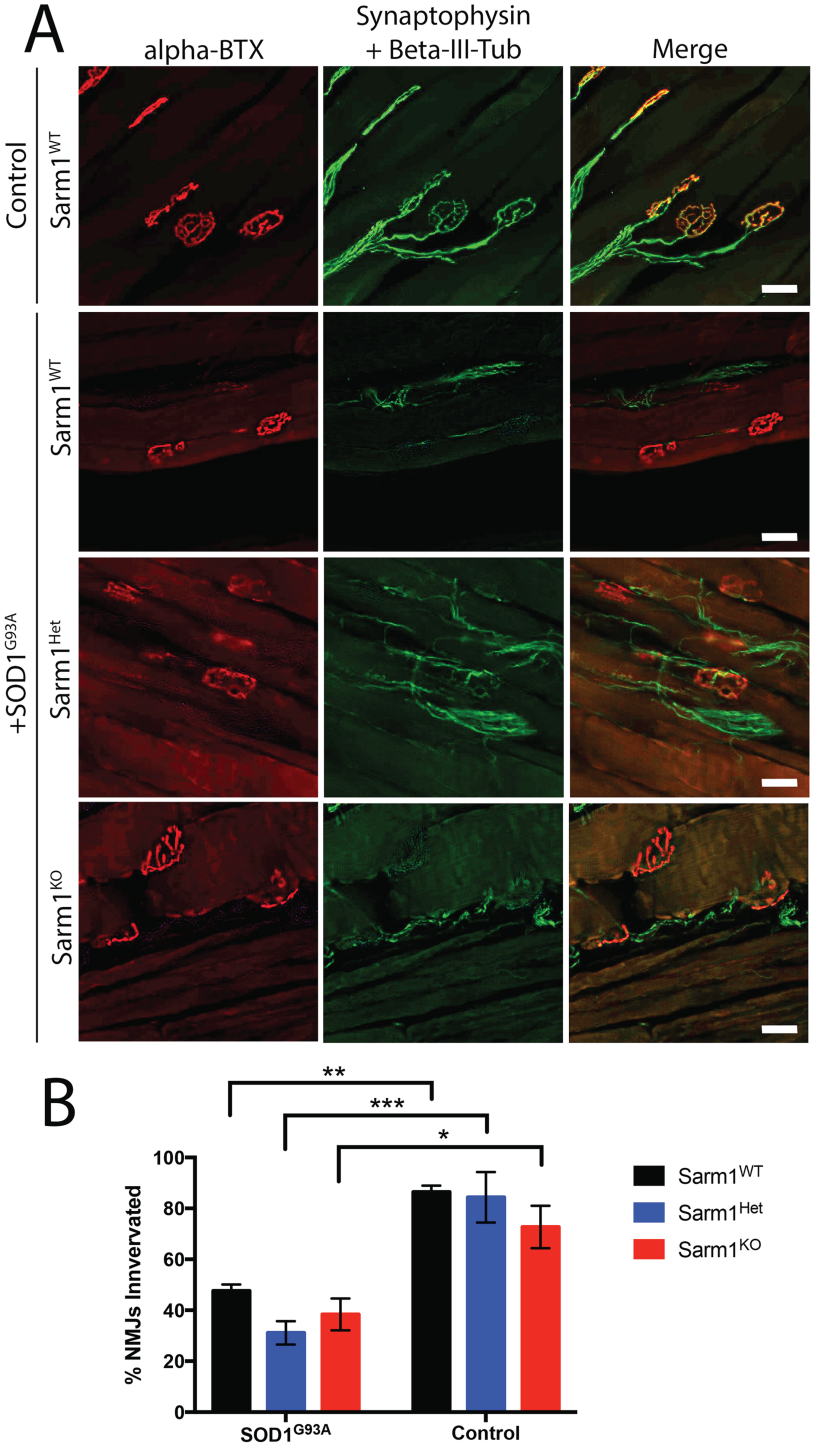
Sarm1 does not contribute to NMJ denervation in SOD1^G93A^ transgenic mice. (**A**) Representative images of immunofluorescent staining of NMJs in the gastrocnemius muscle at p120–130. (Green = synaptophysin and beta-III-tubulin co-stain, Red = alpha-bungarotoxin, scale bar = 50 μm) (**B**) Percentage of innervated NMJs in the gastrocnemius muscles of transgenic cohorts at p120–130 (mean ± SEM, n = 3–5, two-way ANOVA with Bonferonni post-hoc test, *P-value < 0.05, **P-value < 0.01, ***P-value < 0.001).

In addition to driving the programmed degradation of severed axons, Sarm1 also contributes to the destruction of synapses in response to injury. We have previously found that following transection of Sarm1 null mouse peripheral nerves, NMJs corresponding to severed motor axons maintain their innervation of target muscles for several days post-injury (6). As widespread loss of NMJs occurs in ALS, we assessed whether Sarm1 is required for the degeneration of the distal axon and NMJ synapse of SOD1^G93A^ mice. Initial innervation patterns in gastrocnemius muscles in presymptomatic Sarm1^KO^ mice expressing SOD1^G93A^ were comparable to Sarm1^Het^ and Sarm1^WT^ SOD1^G93A^ transgenic animals. Quantification of NMJs at mid to late time points demonstrated progressive loss of intact terminals in all SOD1^G93A^ transgenic cohorts; however, no significant differences were detected between Sarm1^Het^ or Sarm1^KO^ mice at any time point assessed (Fig. 5,S4; p120, n = 3–5; two-way ANOVA: F_Sarm1Genotype_ (2,16) = 1.897, *P*-value = 0.1822; F_SOD1Genotype_ (1,16) = 68, *P*-value < 0.0001). Taken together, our results suggests elimination of Sarm1 signaling is not sufficient to block the progressive destruction of motor neuron axons or their synaptic junctions driven by overexpression of mutant SOD1.

## Discussion

The promotion of regulated axon destruction by Sarm1 presents an appealing target for the treatment of neurodegenerative diseases and injury where pronounced axon degeneration occurs. In the present study we found that genetic suppression of Wallerian-like axon destruction by deletion of Sarm1 neither attenuates axon degeneration nor alters hallmark features of disease progression in the SOD1^G93A^ transgenic mouse model of ALS. Our findings are in line with previous studies of the role of Wallerian-like degeneration in models of motor neuron diseases. Expression of the potent suppressor of Wallerian degeneration Wld^s^ in models of motor neuron degeneration previously yielded varied results, with only modest protection having been reported in SOD1^G93A^ transgenic mice (20). Similarly, the degenerative phenotypes in SOD1^G37R^ or SOD1^G85R^ transgenic mouse models were not changed by the presence of Wld^S^ (21). However, in the *pmn* mouse model of progressive motor neuronopathy, which entails axon degeneration followed by motor neuron death, Wld^S^ significantly suppressed axon loss, rescued motor neuron death and extended animal lifespan (19). These observations validate the notion that treating the axon in some contexts can save neurons, but our data suggest that blocking axon degeneration alone with Sarm1 is not sufficient to ameliorate SOD^G93A^ mutant henotypes. Perhaps the severity and rapid time scale of pathology in SOD1 transgenic rodent models pose a limitation in these studies. Although this model recapitulates the cardinal pathologies of ALS, the potency of high-level overexpression of SOD^G93A^ might potentially mask any modifying effects of Sarm1 on disease progression.

The contribution of Wallerian-like axon degeneration in ALS-associated phenotypes has also been tested in invertebrate models. Loss of Tir-1, the *Caenorhabditis elegans* homolog of Sarm1, delays paralysis induced by expression of mutant of FUS RNA Binding Protein or TAR DNA-Binding Protein 43 (TDP-43) in worms (31). The authors suggest expression of the ALS mutant genes leads to secretion of currently unknown molecules from neurons, activating Tir-1 and the downstream MAP-kinase cascade triggering both an innate immune response and neuron degeneration, though an effect upon neuronal morphology was not reported. In contrast, neither the expression of Wld^S^ nor loss of dSarm or Phr1/Highwire was found to delay morphologically defined degeneration of NMJ in *Drosophila* expressing mutant TDP-43 in motor neurons (32). Taken together these *in vivo* studies in rodent and invertebrate models of ALS argue the molecular pathways promoting Wallerian degeneration, may not be the primary drivers of axon loss or, at a minimum, are functionally redundant with other prodegenerative signaling pathways.

We have found Sarm1 to robustly drive Wallerian axon destruction at both juvenile (Fig. 1B,(6)) and mature adult stages (Fig. 1A), demonstrating that the genes role in this degenerative process is neither restricted to development nor substantially impeded by age. Why, however, inhibition of Sarm1 mediated axon degeneration has such a profound effect on Wallerian degeneration even in neurons at advanced ages but fails to ameliorate ALS-associated neurodegeneration is unclear. Our study demonstrates that in Sarm1 null animals expressing mutant SOD1, Wallerian degeneration of the distal injured axon is impeded (Fig. 1B) but not ALS-associated axonal degeneration (Fig. 4D). Whether any of these models meaningfully recapitulate the molecular mechanisms driving axon loss in patients remains unclear; but if so, our findings suggest the underlying mechanisms may be molecularly distinct.

The role of mitochondria in different forms of neurodegeneration may also be a factor, with evidence suggesting mitochondrial biology contributes to the protective activity of Sarm1 and Wld^S^. Both proteins localize to mitochondria (33–35), with Sarm1 robustly protecting cultured neurons from the downstream reactive oxygen stress caused by mitochondria uncoupling (36). Though there is some evidence of mitochondria abnormalities in SOD1 transgenic mice (30,37), it however remains uncertain whether this is a driving mechanism behind neurodegeneration rather than a secondary response to poor health of the neuron. Interestingly, mitochondria damage in ALS models may in part be due to altered levels of NAD+. The protective activity of both Wld^S^ overexpression and Sarm1 deletion is likely dependent on maintaining neuronal levels of NAD+. Fragmentation of mitochondria seen in cultured motor neurons expressing mutant SOD1 can be rescued by expression of the NAD+-dependent sirtuin SIRT3 (38), suggesting that maintaining high levels of NAD+ may be beneficial to neuronal survival in ALS. However, investigating the neuronal contribution of NAD+ *in vivo* is compounded by its function in maintaining normal glial activity. Depleted levels of NAD+ in cultured astrocytes drives oxidative stress in the cells, subsequently contributing to their toxicity toward co-cultured neurons, an effect that can be rescued by increasing levels of astrocytic NAD+ (39). Further analysis of the role of NAD+ in ALS and other neurodegenerative disease is required to increase our understanding of how this molecule contributes to diverse forms of axon destruction.

Indeed, the process by which motor axons degenerate in ALS does not appear to be Wallerian-like; loss of distal motor axons occurs prior to onset of symptoms in SOD1 transgenic mouse models (40), progressing in a dying back pattern ultimately leading to death of motor neuron cell bodies late in disease (29). In contrast, Wallerian degeneration is characterized by explosive, widespread fragmentation of the axon following severing along its entire length. Interestingly, axonal injury has previously been found to result in exacerbated degeneration of motor neuron cell bodies in the spinal cords of SOD1^G93A^ transgenic mice (43, Neuroscience), suggesting the proximal portion of damaged axons may contain a signaling cascade to enhance destruction of the cell body. Notably, Sarm1 was found to improve acute responses in a mouse model of traumatic brain injury, where crush and shear injuries to axons are more typical (41). We therefore speculate that multiple, potentially parallel mechanisms for axon destruction exist: an acute pathway in which Sarm1 (and an emerging cascade of downstream factors) drives the destruction of severed axons and a second distinct mechanism that occurs as axons undergoing chronic pathology in neurodegenerative disease. This latter pathway may involve Sarm1, but based on our data, we would predict in that case that Sarm1 acts in a redundant fashion with other pathways that are capable of executing axon degeneration even in the absence of Sarm1. Future examination of additional Wallerian degeneration molecules (e.g. Phr1 and Axundead) in these models should clarify this issue. Furthermore, it seems likely that selective protection of axons from degeneration without additionally impeding the destruction of other cellular compartments like the soma might be insufficient to entirely block neurodegeneration in ALS. Notably inhibition of cell body death through deletion of the pro-apoptotic gene Bax, though effective in rescuing cell body death, did not to delay the progression of motor behavior functional decline in SOD1 transgenic mice (30).

Further exploration of the Sarm1 signaling pathway in neurodegeneration is warranted given the ability of Sarm1^KO^ to attenuate axon loss and improve behavioral outcomes in models of Traumatic Brain Injury (TBI) (41) and peripheral neuropathy (44). The role of Sarm1 in promoting axon degeneration may indeed be context-dependent. That the nerve lesioning experiments in our study found Sarm1 to drive axon destruction in both young and aged axons suggests it may still be a viable target for both juvenile (i.e. spinal muscular atrophy) and age-associated neurodegenerative disease (i.e. Parkinson’s, Alzheiemer’s, glaucoma). Until comprehensively tested in the variety of models of neuronal injury and neurodegeneration we will not know the full potential of Sarm1 and its associated axon-destruction cascade as a therapeutic target.

## Materials and Methods

### Animals/scoring

High expression hSOD1^G93A^ (B6.Cg-Tg(SOD1*G93A)1Gur/J) and Sarm1 knockout (B6.129X1-*Sarm1^tm1Aidi^*/J) mice were acquired from Jackson Labs and maintained on a C57Bl6/J backgrounds, in a 12-hr light/dark cycle with *ad libitum* access to food and water. Transgenic colonies were generated by breeding male mice heterozygous for both Sarm1 and SOD1^G93A^ transgene with Sarm1 heterozygous or null females. Mice were scored daily by researchers blinded to genotype for onset of trembling or dragging/paralysis of one or more hind limbs. End-stage mice unable to right within 30 s were euthanized. To encourage feeding, animals approaching end stage had access to wet mash food and gel water packs on their cage floor. For histological experiments, littermate control mice not expressing the SOD1^G93A^ transgene older than p200 were used for comparison to transgenic littermates. Experiments were approved by the University of Massachusetts Medical School Institutional Animal Care and Use Committee.

### Genotyping

All mice were genotyped using standard Polymerase Chain Reaction (PCR) of DNA extracted from ear clippings. DNA was extracted using the DNeasy Blood and Tissue Kit (Qiagen). The following primers were used to amplify DNA for determining genotype: human SOD1 transgene—fwd 5′CAT CAG CCC TAA TCC ATC TGA 3′ and rev 5′TCT TAG AAA CCG CGA CTA ACA ATC 3′; mouse endogenous SOD1—fwd 5′ GCA ATC CCA ATC ACT CCA CAG 3 and rev 5′ GTC CAT GAG AAA CAA GAT GAC 3′ ; mouse endogenous Sarm1—fwd 5′ ACG CCT GGT TTC TTA CTC TAC GA 3′ and rev 5′ GCT GGG GCC TCC TTA CCT CTT 3′; and Sarm1 null neocassette—fwd 5′ CAG GTA GCC GGA TCA AGC GTA TGC 3′ and rev 5′ CCT GTC CGG TGC CCT GAA TGA ACT 3′ (Integrated DNA Technologies). Real-time quantitative PCR was used to estimate human SOD1 transgene copy number. Copy number estimation was calculated based on the difference between the threshold cycles of the hSOD1 transgene compared to an endogenous reference gene, ApoB. Mice with an estimated copy number two standard deviations outside of the average copy number for our colony were removed from the experiment.

### Behavioral testing

Mice were tested weekly with an accelerating rotarod paradigm starting at p50 on a five-station rotarod for mice (Med Associates Inc.). At p50, mice were trained on the rotarod with 30 s at a constant speed of 4 rpm and 30 s at an accelerating rate starting at 4 rpm 1 day prior to the initial rotarod trial. The mice were tested for 3 non-consecutive trials with the rotarod accelerating from 4 rpm to 40 rpm for a maximum trial of 300 s. Symptomatic mice that were unable to perform on the rotarod were scored with a 0. Forelimb and all-limb grip strength was measured weekly using a digital force gauge (Mark-10, USA). Each mouse was tested five consecutive times with the highest measurement recorded in newtons (N).

### MUNE

MUNE recordings were carried out as described previously (27,42). Briefly, mice were anesthetized through inhalation of isofluorane, maintained throughout the procedure via a nose cone. A single hind limb was shaved, wiped with alcohol and restrained on a Styrofoam board using adhesive tape. A recording ring electrode (CareFusion) was coated with electrode gel (SignaGel) and placed over the gastrocnemius muscle, a reference electrode over the tendon and a grounding surface electrode (CareFusion) on the tail. Disposable monopolar 28G needle electrodes were used for stimulation. The stimulating cathode was placed 5 mm proximal to the recording ring electrode, with the anode placed subcutaneously at the midline over the sacrum.

All electrophysiological recordings were made using a portable electrodiagnostic system (Cardinal Synergy). For MUNE and motor conduction studies the following settings were used: low-pass filter, 30 Hz; high-pass filter, 10 kHz; and Squarewave pulses of 0.1-ms duration. Supramaximal responses were gradually generated (typically <10 mA). The distance between distal and proximal stimulation sites was recorded. The distal latency, distal and proximal CMAP amplitudes, distal and proximal CMAP durations (measured from onset of initial negative deflection to initial return to baseline), and conduction velocity were determined for each nerve studied.

The low-pass filter was set at 20 Hz, and the high-pass filter was set at 10 kHZ. Data were acquired with a sensitivity of 100 μV per division and sweep speed of 1 ms per division. Using a repetition rate of 1 per second, stimulus intensity was increased and a single motor unit response was produced. Stimulation was further increased, and quantal increases in the response were recorded. Individual motor unit amplitude of was calculated as the mean of five quantal increases. MUNE was calculated from the CMAP maximal amplitude divided by the mean amplitude of surface-detected motor unit action potential. After recording animals were euthanized by inhalation of isofluorane.

### NMJ histology

Mice were euthanized via isofluorane inhalation and transcardially perfused for 2 min with Phosphate-buffered saline (PBS), followed by 5 min with 4% paraformaldehyde. The gastrocnemius muscles were dissected and fixed overnight in 1.5% paraformaldehyde and then transferred to a 25% sucrose solution overnight (4°C). After sinking, the muscles were embedded in Optimal Cutting Temperature (OCT) compound and 35 μm sections collected on a cryostat (Leica), frozen on slides and stored at −80°C. To stain for NMJs, slides were thawed completely and allowed to dry before being washed with PBS, followed by PBS with 0.4% triton-X100. The muscle sections were blocked with 10% donkey serum (EMD Millipore) in 0.4% triton-X100 PBS and then incubated overnight with a 1:5 dilution of rabbit anti-synaptophysin (Invitrogen) and a 1:1000 dilution of rabbit anti-neuronal class III beta-tubulin (Covance) primary antibodies in blocking solution. The sections were washed with PBS and then incubated overnight with 1:500 donkey anti-rabbit secondary antibody conjugated with alexa-488 (Life Technologies) and 1:500 alpha-bungarotoxin conjugated with 555 nm fluorophore (Thermo Fisher Scientific, USA) diluted in PBS. The sections were washed with PBS and coverslips were mounted with Shandon Immunomount (Fisher Scientific). NMJs were imaged on a Nikon Eclipse Ti and scored for innervation status by two investigators blinded to genotype.

### Sciatic nerve axotomy

Assessment of Wallerian degeneration by lesioning of the sciatic nerve was carried out as described previously (6). Briefly, mice were anesthetized by inhalation of isofluorane, the skin was shaved and cleaned with ethanol and povidone-iodine. A 5 mm incision was made to the skin and the sciatic nerve was exposed. The sciatic nerve was grasped with fine forceps, and a 1–2 mm portion of nerve was removed, ensuring that the distal portion of nerve was lesioned completely. The incision was sutured, the mouse was treated with appropriate analgesia and returned to the home cage to recover. Mice were maintained for 14 days post-axotomy, subsequently euthanized, and the sciatic nerve was processed for semi-thin sectioning. The contralateral, intact sciatic nerve was also dissected from each animal as an internal, uninjured control.

### Nerve semi-thin sectioning and toluidine blue histology

Sciatic nerves and L5 ventral nerve roots were dissected from 4% paraformaldehyde perfusion fixed mice. Following dissection nerves were immersion fixed overnight at 4°C in 2.5% gluteraldehyde in 0.1 M cacodylate buffer (pH 7.2). The following day nerves were washed in cacodylate buffer and subsequently post-fixed in 1% osmium tetroxide at room temperature for 1 hr. Following washing with dH_2_O, nerves were dehydrated through a graded ethanol series of 0–100% in 20% increments, followed by two changes of 100% ethanol. Nerves underwent resin infiltration through two changes of 100% propylene oxide followed by overnight incubation in a solution of 50%SPI-Pon 812 resin in propylene oxide. Samples were then rinsed with SPI-Pon 812 resin and polymerized in SPI-Pon 812 at 68°C in flat embedding molds. Semi-thin sections (0.6 μm) were collected using an ultramicrotome (Reichert–Jung UltraCut E) fitted with a diamond knife (Ultra 45^o^, DiATOME), mounted onto glass slides and stained with 1% toluidine blue. Images were captured using a 63× lens on a Nikon Ti-E inverted widefield microscope and stitched using Velocity software (3i). Diameters of regularly shaped healthy axons were measured using ImageJ (National Institutes of Health). Images were thresholded and particles of circularity 0.5–1 and minimum diameter 0.5 μm were analyzed. Incorrectly identified objects were manually removed from the data set. Minimum diameter was used for statistical analysis. For total axons counts, healthy, irregularly shaped axons not detected by particle analysis were manually added to the data set.

### Experimental design and statistical analysis

Experimental data were conducted by researchers blinded to the genotype of animals. All statistical analyses were conducted, and graphs were plotted using Graphpad Prism (GraphPad Software, Inc.). Unless stated otherwise, all charts show mean ± SEM. Statistical test used are described in the relevant results or figure legends. Tests used are unpaired *t* test, one-way ANOVA and two-way ANOVA with Bonferroni post-hoc testing. *P*-values < 0.05 were considered significant for all statistical analyses used.

## Supplementary Material

Supplementary Data
