# Supplementary material for: Loss of Sarm1 does not suppress motor neuron degeneration in the SOD1G93A mouse model of amyotrophic lateral sclerosis
Source: Hum Mol Genet. 2018 Jul 14;27(21):3761–71. doi: 10.1093/hmg/ddy260 (PMC6196650; doi:10.1093/hmg/ddy260)
Supplement: Supplementary Data [file ddy260_supp.zip › SOD1-Sarm Supplementary Materials CORRECTED FIGURE S2 18-7-18.pdf]

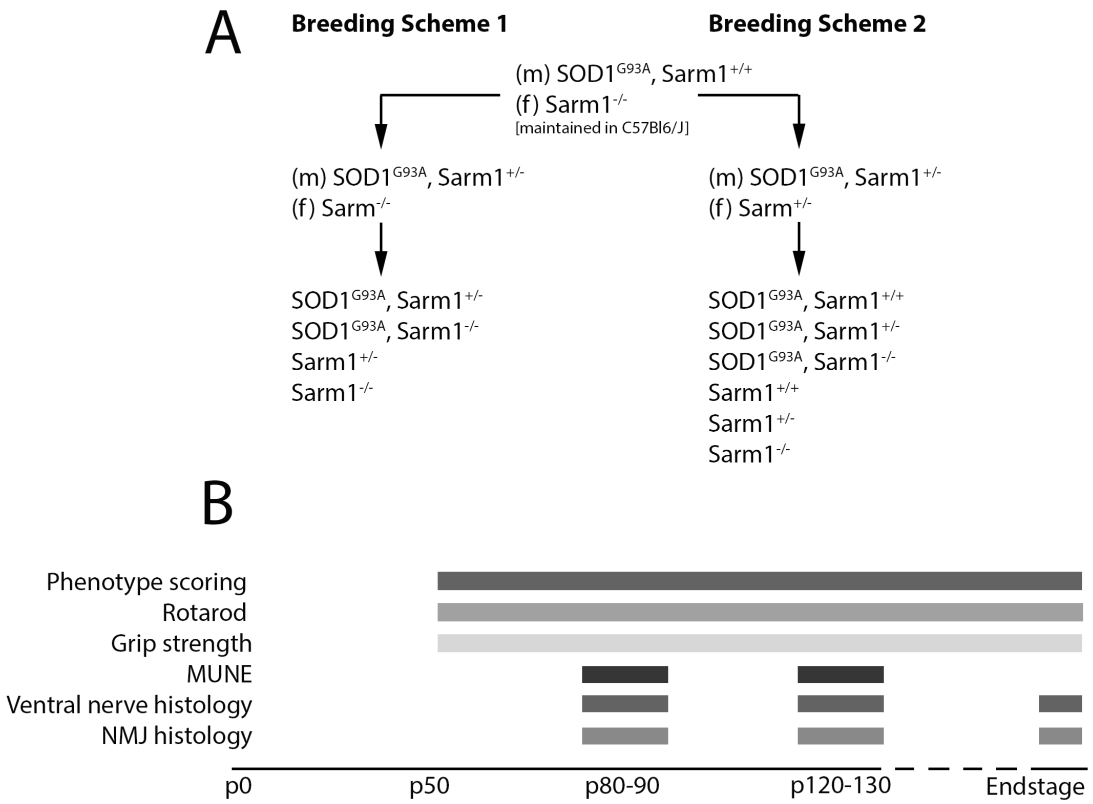

Figure S1. Experimental design for Sarm1 & SOD1^{G93A} transgenic mouse studies. Breeding schemes (A) and timeline (B) of experiments for analysis of SOD1^{G93A} Sarm1 transgenic cohorts.

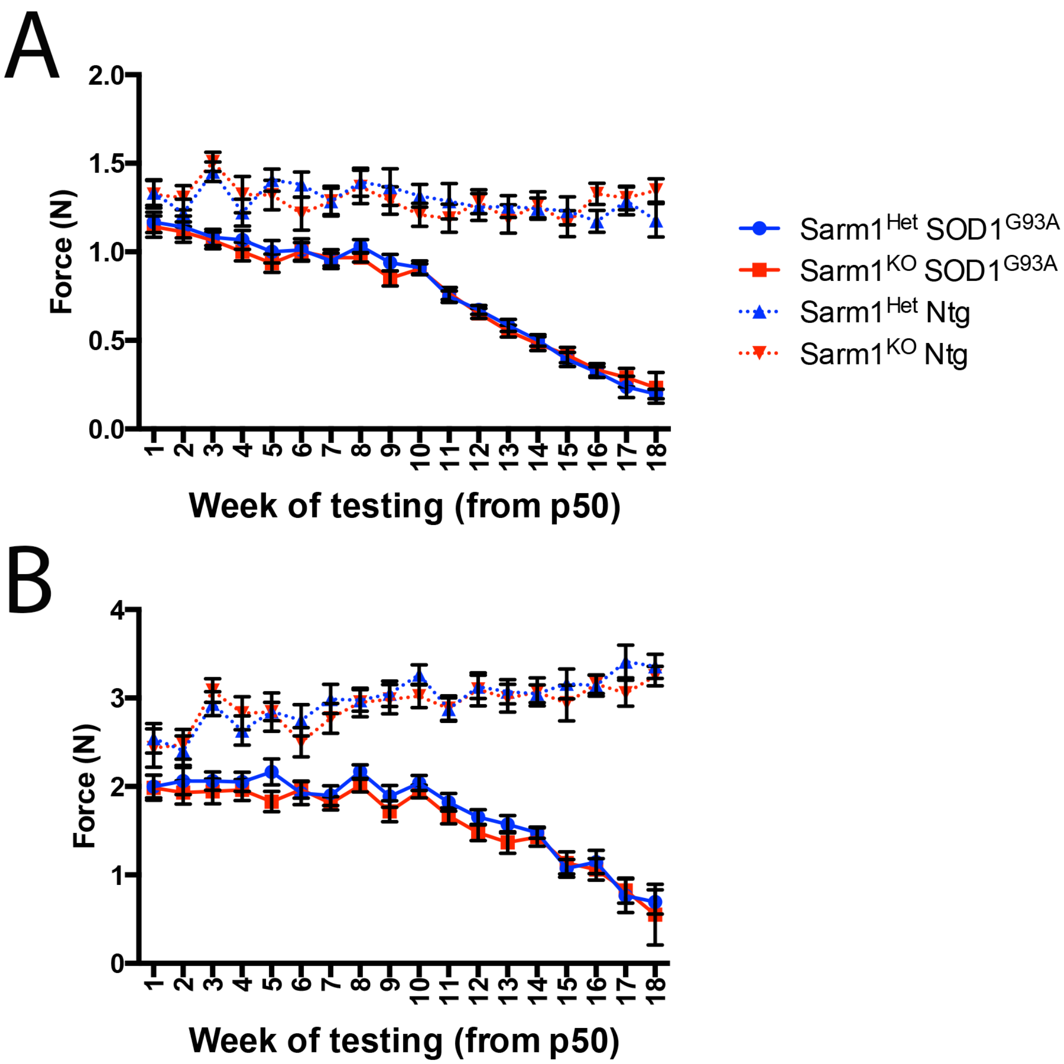

Figure S2. Progressive decline in grip strength of SOD1^{G93A} transgenic mice is not ameliorated in Sarm1 null animals. Weekly grip strength testing of (A) fore limb only and (B) all limbs of Sarm1 null cohorts (mean±SEM, SOD1^{G93A} groups n=20, control groups n=15, two-way ANOVA with Bonferonni post-hoc test).

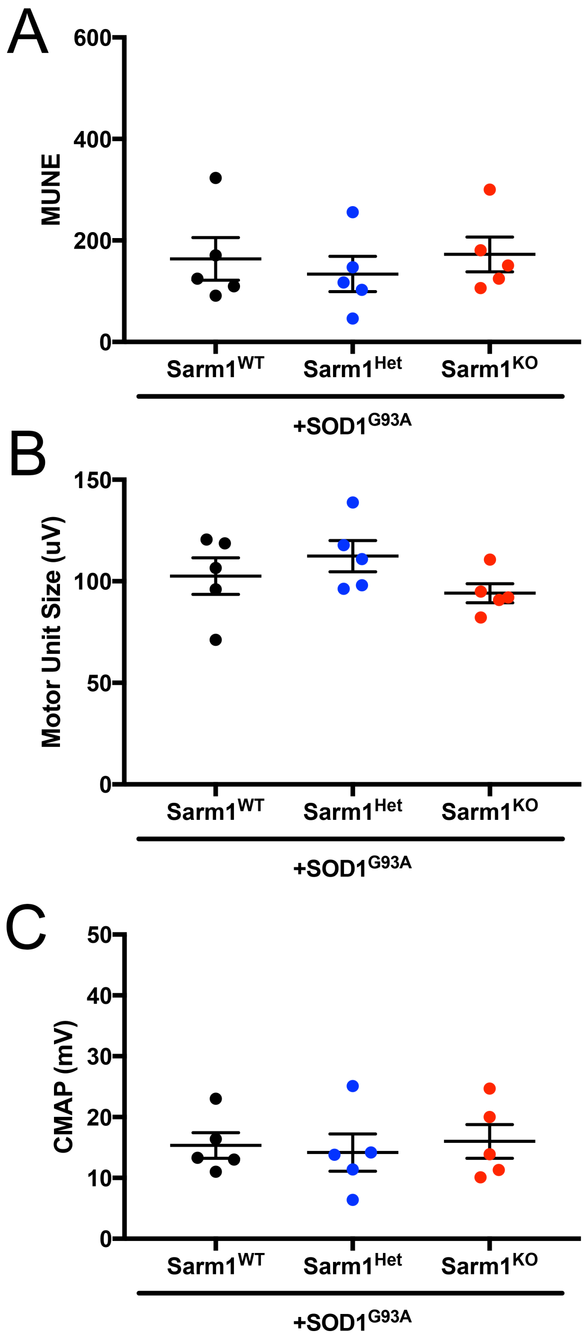

Figure S3. Sarm1 does not contribute to electrophysiological deficits in the early stages SOD1^{G93A} symptomatic onset. *In vivo* electrophysiological recording of gastrocnemius neuromuscular function in SOD1^{G93A} transgenic mice either wild (black) heterozygous (blue) or null (red) for Sarm1. Assessment at the early symptomatic p80-90 time point found no significant difference in (A) motor unit number estimation (MUNE), (B) motor unit size and (C) compound motor action potential (CMAP) of transgenic cohorts (mean±SEM, n=5, one-way ANOVA).

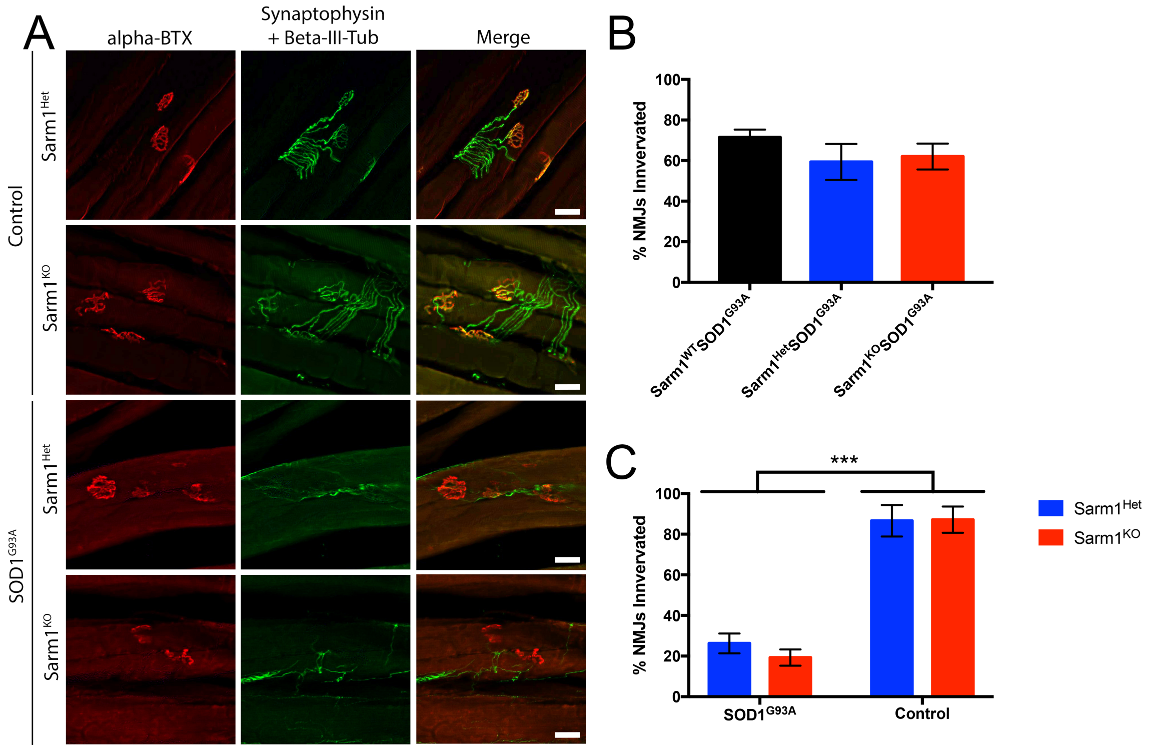

Figure S4. SOD1^{G93A} induced denervation of neuromuscular junction endplates is not alleviated in Sarm1 null mice. Representative images showing neuromuscular junctions of SOD1^{G93A} or control end stage Sarm1 heterozygous or null mice, showing denervation of endplates in transgenic cohorts (Green=synaptophysin and beta-III-tubulin co-stain, Red = alpha-bungarotoxin, scale bar = 50μm). Percentage of normally innervated neuromuscular junctions in the gastrocnemius muscles of transgenic cohorts at (B) p80-90 (mean±SEM, n=5, one-way ANOVA) and (C) endstage (mean±SEM, n=3-5, two-way ANOVA, *** $p<0.001$).
